# IMPROVING SERVICES TO LGBTQ+ OLDER ADULTS: NEEDS OF MICHIGAN LONG-TERM CARE PROVIDERS

**DOI:** 10.1093/geroni/igac059.196

**Published:** 2022-12-20

**Authors:** Anne Hughes, Linda Keilman, Leo Kattari

**Affiliations:** Michigan State University, East Lansing, Michigan, United States; Michigan State University, East Lansing, Michigan, United States; Michigan State University, East Lansing, Michigan, United States

## Abstract

Long term care (LTC) providers need information about providing competent care to LGBTQ+ older adults. Providers who are not educated can inadvertently provide biased care. Our research identified preferences for education among providers of LTC services in Michigan (MI). In this descriptive cross sectional study we used an online survey to collect data from MI facilities (N= 429). Survey items included facility characteristics, diversity training history, perceived need for training on LGBTQ+ older adults, barriers to training, interest in additional training on LGBTQ+ older adults and LTC, and training preferences. Results were obtained from 71 facilities. Thirty-seven percent of responses came from direct care workers, 63% from administrators. There was good support for diversity training, with 24% stating diversity training was “somewhat important” and 74% stating it was “very important”. A majority (63%) had had some diversity training in the past year. Most (72%) endorsed the need and desire for more training on LGBTQ+ aging. More content on transgender older adults and concerns such as room assignments, dementia, and use of pronouns were identified. Barriers to training included: cost, availability of trainers with the appropriate expertise, ability to reach large numbers of employees, staff turnover, bias and ignorance among staff and residents, and need to provide rationale for this type of training. Most endorsed a mixed type of training and a training length between 1 and 3 hours. Diversity training is critical to LTC and needs to be expanded to include needs of the aging LGBTQ+ community.

